# Elevated serum uric acid is associated with cognitive improvement in older American adults: A large, population-based-analysis of the NHANES database

**DOI:** 10.3389/fnagi.2022.1024415

**Published:** 2022-12-08

**Authors:** Rulin Geng, Yu Zhang, Miao Liu, Shengfeng Deng, Jingwen Ding, Hongfei Zhong, Qiuyun Tu

**Affiliations:** Department of Geriatrics, The Fifth Affiliated Hospital of Sun Yat-sen University, Zhuhai, China

**Keywords:** cognitive function, dementia, Alzheimer’s disease, serum uric acid, NHANES

## Abstract

**Background:**

The many studies revealing a connection between serum uric acid (SUA) and dementia have reported conflicting results. This study sought to investigate the relations between SUA and cognitive function in older adults.

**Materials and methods:**

The sample was 2,767 American adults aged ≥60 years from the National Health and Nutrition Examination Survey 2011–2014. Cognitive performance was evaluated by the Consortium to Establish a Registry for Alzheimer’s Disease test, animal fluency test, digit symbol substitution test, and composite z-score. Multivariate linear regression analyses were conducted to estimate the association between SUA and cognitive function.

**Results:**

SUA level and cognitive function were significantly, positively correlated. Age significantly correlated with the association between SUA and cognitive function.

**Conclusion:**

These findings support a connection between SUA and cognition, showing a positive link between SUA and cognitive scores among older American adults. We contend that a slight rise in uric acid within the normal range is advantageous for enhanced cognition. To confirm the precise dose-time-response relation, more tests will be needed.

## Introduction

As life expectancy increases, age-related cognitive decline may become a significant health challenge for the older adult population (Alzheimer's., 2021), and cognitive impairment has emerged as an important public health concern for the aging population in the United States (Olivari et al., 2020). Cognitive impairment refers to an individual’s struggle to remember things, learn new information, focus, or make decisions that impact their daily lives (Cognitive Disorders, 2011). Mild cognitive impairment (MCI), Alzheimer’s disease (AD), vascular dementia (VAD), and other dementia types are examples of cognitive impairment that can range from mild to severe. A person with severe cognitive impairment may lose their memory, comprehension, and ability to read, speak, or write, making it impossible for them to live independently long-term. The most prevalent form of dementia is AD, which affects 6.2 million Americans aged 65 and older. With a reported rise in AD fatalities of more than 145% during the previous 10 years, this number may reach 13.8 million by 2060(Alzheimer's., 2021). There is currently no effective dementia treatment, and cognitive decline is progressive and irreversible. Memantine and cholinesterase inhibitors are the only medications with clinical evidence supporting their treatment of cognitive impairment symptoms (Vaz and Silvestre, 2020). However, long-term donepezil and memantine treatments offer minimal benefit for patients with mild-to-severe dementia (Howard et al., 2015). Therefore, it is essential to prevent the risk factors that cause cognitive decline, to avoid the development of dementia.

Serum uric acid (SUA) is the byproduct of purine metabolism and is affected by diet and kidney function (Estiverne et al., 2020). Some studies suggest that SUA has a preventive effect as an antioxidant (Yeung et al., 2019) against cognitive impairment (Lu et al., 2016; Tana et al., 2018; Lee et al., 2021b) and neurological disorders (Huang et al., 2018; Zhang et al., 2018). Elevated SUA is frequently observed in patients with gout or hyperuricemia. Hyperuricemia, defined as SUA >420 μmol/L (7 mg/dl). Though many studies point to a connection between SUA and dementia or cognitive decline, their findings are contradicted. According to a cohort study by Lu et al. (2016), gout is negatively correlated with the probability of developing AD. Using fluorodeoxyglucose-positron emission tomography scans of 979 participants, Lee et al. revealed that higher SUA levels indicated a protective trend among those with cognitive impairment (Lee et al., 2021b). Additionally, higher SUA level was associated with lower incidence of MCI in a prospective cohort of 3,103 older adults (Chen et al., 2021). Cumulatively, these studies indicate that higher SUA levels may have neuroprotective effects, while lower levels may increase the risk of dementia (Xue et al., 2017). In contrast, a causal link between gout and AD was unsupported by a Mendelian randomized analysis (Yuan and Yang, 2018; Lee, 2019). Further, Lee et al. (2021b) also discovered that increased SUA levels harm cognitive function in healthy adults.

Herein, a representative sample of older adults who participated in the National Health and Nutrition Examination Survey (NHANES) were used to examine the connection between blood SUA and cognition.

## Materials and methods

### Data collection and study population

The NHANES is a cross-sectional survey conducted every 2 years to evaluate the nutritional and physical health of adults and children in the United States. NHANES is a significant program of the National Center for Health Statistics (NCHS), a division of the Centers for Disease Control and Prevention, which is responsible for providing critical health statistics (National Health and Nutrition Examination Survey, 2017a). The NCHS Research Ethics Review Board approved the NHANES program, and all survey participants provided written informed consent (National Health and Nutrition Examination Survey, 2017e).

**Figure 1:**
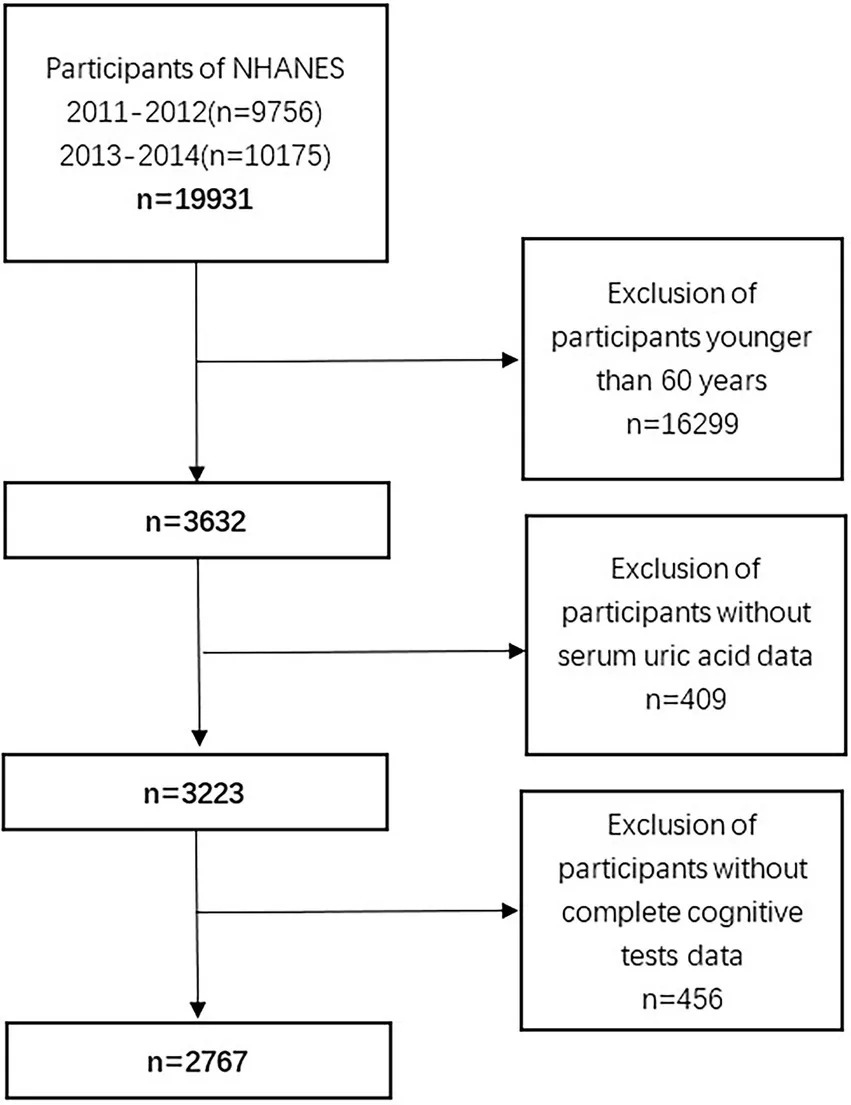
Flow chart of the procedure for selecting eligible participants.

For these analyses, we used data from 2011 through 2014, which corresponds to two NHANES cycles. During these periods, 19,931 Americans participated; however, we only included 3,632 individuals aged ≥60 years. After removing those with missing SUA data (*n* = 409) and incomplete cognitive tests (*n* = 456), a total of 2,767 participants were available for analyses (Figure 1).

### Variables

The study exposure variable was SUA and the outcome was cognitive function. The latter was evaluated using the Consortium to Establish a Registry for Alzheimer’s Disease (CERAD) test, the animal fluency (AF) test, and the digit symbol substitution test (DSST). We used the following factors as covariates in our analyses: age, gender, race, level of education, marital status, ratio of income to poverty, difficulties with remembering or thinking, albumin (ALB), blood urea nitrogen (BUN), creatinine, body mass index (BMI), diabetes, heart failure, stroke, hypertension, smoking, alcohol consumption, and physical activity. Public access to these comprehensive data can be found at http://www.cdc.gov/nchs/nhanes/.

#### Cognitive performance assessment

Participants aged ≥60 years underwent cognitive tests for the NHANES survey from 2011 to 2014. The NHANES Mobile Examination Center-employed interviewers were qualified to conduct the evaluations, including: (1) CERAD word learning and recall modules; (2) the AF test; and (3) the DSST (National Health and Nutrition Examination Survey, 2017b). These assessments are commonly used to evaluate memory, language, attention, learning capacity, processing speed, and executive function in older adults in large-scale screening, epidemiological, and therapeutic research (Fillenbaum et al., 2008; Cukierman-Yaffe et al., 2020; Golub et al., 2020; Ma et al., 2020; Lucey et al., 2021; Wang et al., 2021; Lee et al., 2021a).

The CERAD word learning test measures immediate and delayed learning of new linguistic information. It consists of three consecutive word learning trials and delayed recall (i.e., memory subdomains; Fillenbaum et al., 2008). For the learning trials, participants read aloud a list of 10 unrelated words, then recall as many as possible. The delayed word recall is assessed 8–10 mins after the trial begins. For each trial, the words are presented in a different order. A maximum score of 10 points for each trial can be earned by correctly reciting each word. Three learning trials and one delayed recall trial make up the overall CERAD test score (National Health and Nutrition Examination Survey, 2017b), for a highest possible score of 40.

Verbal fluency was examined using the AF test (Rofes et al., 2020), which can differentiate between those with normal cognitive function, those with MCI, and those with more severe forms of cognitive impairment (e.g., AD; McDonnell et al., 2020). Points are given for the number of animals recalled in 1 min (National Health and Nutrition Examination Survey, 2017b).

The DSST is the Wechsler Adult Intelligence Scale (version III) performance module that relies on processing speed, sustained attention, and working memory (Wechsler, 1997). For this assessment, the participant uses a paper table with nine number–symbol pairs, and has 2 mins to copy the symbols into 133 boxes adjacent to numbers. The total number of accurate matches determines the score (National Health and Nutrition Examination Survey, 2017b).

We summed the z-scores from each of these tests, for a composite score representing holistic cognitive capacity: (individual test score − mean score)/standard deviation (SD; Fan et al., 2021). Higher scores on all exams indicate superior cognitive performance. A standard criterion for low cognitive performance is not currently established for the CERAD, AF, or DSST tests. Therefore, based on methods previously published (Chen et al., 2017; Brody et al., 2019), we set the cutoff at the 25th percentile, or lowest quartile, for each. These values were 20 for CERAD cognitive performance, 12 for the AF test, 32 for DSST, and −1.7 for the composite z-score. Participants were then split into two groups based on this threshold: normal cognitive performance and low cognitive performance.

#### Serum uric acid

SUA data were obtained from two nationally representative NHANES cycles, 2011–2012 and 2013–2014. The DxC800 used a timed endpoint approach to detect SUA levels (National Health and Nutrition Examination Survey, 2017f). In the subsequent regression analysis, SUA was separated into four groups by quartiles, with Q1 serving as the reference group (Q1: 178.4–279.5 μmol/L; Q2: 279.6–333.0 μmol/L; Q3: 333.1–392.5 μmol/L; Q4: 392.6–701.9 μmol/L).

#### Covariates

We included pertinent covariates in the analysis based on prior research demonstrating their links to SUA (Dehlin et al., 2020; Dong et al., 2021; Nieradko-Iwanicka, 2022), cognitive impairment (Stephen et al., 2017; Rehm et al., 2019; Jia et al., 2020), or both (Chen et al., 2021). We gathered several potential confounders, such as age (60–69 years, 70–79 years, ≥80 years), gender (male and female), race (Mexican American, other Hispanic, white, Black, Asian, and other race[s]), education (less than high school, high school, above high school), marital status (never married, married, widowed, divorced), ratio of income to poverty (≤1.99 and ≥2), difficulties with remembering or thinking, ALB, BUN, creatinine, BMI, diabetes, heart failure, stroke, hypertension, smoking, alcohol consumption, and physical activity. The ratio of family income to poverty was used to define income (National Health and Nutrition Examination Survey, 2017c). The participants’ self-cognition rating is represented by difficulties with remembering or thinking. BUN and smoking did not differ significantly between cognitive groups in our analyses (*p* > 0.05, Table 1) Difficulties with remembering or thinking was not associated with SUA (*p* > 0.05). The three factors were hence left out of model adjustment.

**Table 1:** Weighted study population characteristics based on cognitive grouping.

	Total	CERAD			AF			DSST			Composite z-score		
		Normal Cognitive Performance	Low Cognitive Performance	P value	Normal Cognitive Performance	Low Cognitive Performance	P value	Normal Cognitive Performance	Low Cognitive Performance	P value	Normal Cognitive Performance	Low Cognitive Performance	P value
N	2,767	2092	675		2,119	648		2,130	637		2078	689	
Age (%)				<0.01			<0.01			<0.01			<0.01
60–-69 years	56.77	62.4	32.62		61.23	33.18		60.49	31.32		61.81	28.69	
70–-79 years	29.28	27.58	36.59		27.35	39.49		28.36	35.6		28.3	34.76	
≥80 years	13.95	10.02	30.8		11.42	27.33		11.16	33.08		9.89	36.55	
Gender (%)				<0.01			0.28			0.8			0.29
Male	46.36	44.08	56.14		46.81	44		46.27	46.97		45.94	48.71	
Female	53.64	55.92	43.86		53.19	56		53.73	53.03		54.06	51.29	
Race (%)				<0.01			<0.01			<0.01			<0.01
Mexican American	3.37	2.96	5.14		3.15	4.55		2.53	9.13		2.76	6.77	
Other Hispanic	3.5	2.97	5.77		2.98	6.24		2.27	11.92		2.43	9.43	
White	80.41	81.72	74.78		83.3	65.09		84.12	54.98		83.75	61.75	
Black	7.89	7.56	9.3		6.24	16.58		6.07	20.35		6.33	16.56	
Asian	3.2	3.23	3.08		2.73	5.69		3.22	3.02		3.06	4	
Other Race	1.64	1.57	1.95		1.6	1.85		1.79	0.61		1.66	1.49	
Education (%)				<0.01			<0.01			<0.01			<0.01
Below high school	15.42	12.34	28.63		12.41	31.36		10.63	48.25		11.33	38.28	
High school	22.17	20.83	27.9		20.7	29.94		21.77	24.86		20.84	29.58	
Above high school	62.41	66.83	43.48		66.89	38.7		67.6	26.89		67.84	32.14	
Marital status (%)				<0.01			<0.01			<0.01			<0.01
Married	65.26	66.61	59.47		67.25	54.71		68.07	46		67.55	52.51	
Widowed	16.53	14.85	23.74		14.47	27.45		14.3	31.86		14.49	27.91	
Divorced	13.81	14.19	12.18		13.94	13.1		13.52	15.82		13.85	13.58	
Never married	4.4	4.35	4.61		4.33	4.74		4.11	6.32		4.11	6	
Ratio of family income to poverty (%)				<0.01			<0.01			<0.01			<0.01
≤1.99	31.93	27.87	49.35		28.48	50.58		26.8	67.9		27.6	56.65	
≥2	68.07	72.13	50.65		71.52	49.42		73.2	32.1		72.4	43.35	
Difficulties remembering or in thinking (%)	12.64	9.69	25.3	<0.01	10.19	25.6	<0.01	10.36	28.27	<0.01	9.17	31.98	<0.01
ALBA (g/L)	42.11 ± 2.91	42.18 ± 2.87	41.81 ± 3.04	<0.01	42.23 ± 2.81	41.48 ± 3.30	<0.01	42.26 ± 2.75	41.06 ± 3.64	<0.01	42.24 ± 2.79	41.38 ± 3.42	<0.01
BUN (mmol/L)	5.87 ± 2.67	5.91 ± 2.68	5.70 ± 2.62	0.12	5.88 ± 2.68	5.80 ± 2.60	0.55	5.88 ± 2.66	5.79 ± 2.75	0.57	5.88 ± 2.65	5.79 ± 2.74	0.51
Creatinine (μmol/L)	87.82 ± 51.82	85.36 ± 48.89	98.38 ± 61.78	<0.01	85.79 ± 39.70	98.54 ± 91.76	<0.01	84.18 ± 29.18	112.73 ± 120.61	<0.01	84.41 ± 32.41	106.83 ± 106.68	<0.01
SUA (μmol/L)	333.79 ± 84.97	332.33 ± 84.71	340.06 ± 85.77	0.06	333.54 ± 84.68	335.12 ± 86.43	0.72	332.28 ± 84.09	344.10 ± 90.09	0.01	333.22 ± 85.33	336.96 ± 82.81	0.41
BMI (Kg/M^2)	29.05 ± 6.26	29.25 ± 6.33	28.18 ± 5.87	<0.01	29.09 ± 6.19	28.82 ± 6.60	0.41	29.04 ± 6.22	29.14 ± 6.55	0.77	29.18 ± 6.28	28.28 ± 6.07	<0.01
Diabetes (%)	18.91	18.4	21.1	0.36	17.7	25.29	<0.01	17.42	29.1	<0.01	17.03	29.41	<0.01
Heart failure (%)	6.74	5.59	11.66	<0.01	5.91	11.09	<0.01	5.51	15.16	<0.01	5.74	12.31	<0.01
Stroke (%)	6.46	5.59	10.2	<0.01	5.54	11.37	<0.01	5.28	14.54	<0.01	5.44	12.13	<0.01
Smoking (%)	22.5	22.11	24.26	0.46	22.11	24.42	0.44	21.68	28.05	0.06	21.98	25.26	0.29
Hypertension (%)	58.48	56.46	67.2	<0.01	56.74	67.66	<0.01	56.55	71.69	<0.01	56.46	69.72	<0.01
Alcohol consumption (%)	73.15	74.63	66.73	<0.01	74.79	64.4	<0.01	75.35	57.62	<0.01	75.43	60.19	<0.01
Physical activity (%)	61.91	64.6	50.4	<0.01	65.69	41.87	<0.01	65.29	38.79	<0.01	65.5	41.92	<0.01

Mean ± SD for continuous variables: weighted linear regression model-calculated P value. (%) for categorical variables: the P value was calculated by the weighted chi-square test. ALB: Albumin; BMI: Body mass index; BUN: Blood urea nitrogen; CERAD: Consortium to Establish a Registry for Alzheimer’s Disease; AF: Animal fluency test; DSST: Digit symbol substitution test.

### Statistical analysis

Sample weights were modified when combining the data, following NHANES analysis guidelines and tutorials (original 2-year sample weights/2) (National Health and Nutrition Examination Survey, 2017d).

As noted above, we divided participants into normal and low cognitive performance groups, divided SUA levels into four (quartile-based) groups for categorical analyses, and chose well-established cognitive performance and/or SUA-associated factors. Confounding variables were not accounted for in Model 1. Age, gender, and race adjustments were made in Model 2. The Model 2 factors plus education, marital status, poverty to income ratio, ALB, creatinine, BMI, diabetes, heart failure, stroke, hypertension, alcohol consumption, and physical activity were adjusted for in Model 3.

The study population characteristics are reported as mean ± SD for continuous variables and percent for categorical variables. The chi-square test and variance analyses were applied to categorical and continuous variables, respectively, to identify between-group differences. A multifactor weighted linear regression model was used to assess the association between SUA and cognitive function. We conducted hierarchical multiple regression analyses for age, sex, and racial subgroups to see if the association between SUA and cognitive performance was correlated with these demographic characteristics. To ascertain whether any measure was responsible for a statistically significant portion of variance, we also conducted interactive analyses. We used the regression coefficient β value and 95% confidence interval (CI) to describe these results. Values of *p* < 0.05 were deemed statistically significant.

The nonlinear relation between SUA and cognitive function was also evaluated using a generalized additive model and smooth curve fitting. The relation between SUA and cognitive function was calculated using a recursive algorithm for the nonlinear model. Two linear regression models were established on either side of the inflection point to identify nonlinearity. Interaction analyses were conducted using Stata 17.0 (Stata Corporation, College Station, TX). The R package^1^ and Empower Stats^2^ were used for other analyses.

## Results

### Study population

As shown in Table 1, a total of 2,767 participants aged 60–80 years were included herein, split into normal and low cognitive performance groups using the 25th percentile threshold of cognitive performance described above. Participants were around age 70 years; 53.64% were women, 80.41% were non-Hispanic white, 62.41% had a college degree or higher, 65.26% were married, 31.93% had incomes at or below the poverty line, and 12.64% reported memory or cognitive impairments. The cognitive performance groups differed significantly (*p* < 0.05) on age, gender, race, education, marital status, the ratio of family income to poverty, difficulties with remembering or thinking, ALB, creatinine, SUA, BMI, diabetes, heart failure, stroke, hypertension, alcohol consumption, and physical activity. Neither BUN nor smoking differed between the groups.

More participants who reported poorer cognitive performance were age ≥ 70 years, were non-white, had a high school or lower education, were widowed, had lower income, and had worse ratings of their own cognition. The poor cognitive function group also had lower rates of alcohol consumption and physical activity, as well as greater incidence of diabetes, heart failure, stroke, and hypertension. They also had higher BMI, increased creatinine, and reduced serum ALB levels. Between age groups, all cognitive scores differed significantly, and as participants aged, the chance of cognitive impairment increased.

### Association between SUA and cognitive function

Table 2 depicts the association between blood SUA and the various cognitive scores. SUA was significantly, negatively correlated with cognition in the unadjusted model (Model 1; CERAD β = −0.0043, 95% CI [−0.0071, −0.0016], *p* < 0.01; DSST β = −0.0160, 95% CI [−0.0233, −0.0087], *p* < 0.01; composite z-score β = −0.0018, 95% CI [−0.0028, −0.0007], *p* < 0.01). In those with SUA levels from 392.6–701.9 μmol/L, compared with those with lower levels, SUA category was significantly and inversely associated with CERAD, DSST, and composite z-score (CERAD β = −1.0583, 95% CI [−1.7305, −0.3861], *p* < 0.01; DSST β = −2.3357, 95% CI [−4.0994, −0.5720], *p* < 0.01; composite z-score β = −0.3005, 95% CI [−0.5556, −0.0454], *p* < 0.05). There were also significant between-SUA quartile group differences (*p* < 0.05). In model 3, except for CERAD, SUA and cognitive scores were significantly correlated (AF β = 0.0028, 95% CI [0.0002, 0.0055], *p* < 0.05; DSST β = 0.0070, 95% CI [0.0005, 0.0135], *p* < 0.05; composite z-score β = 0.0014, 95% CI [0.0004, 0.0023], *p* < 0.01). The highest SUA quartile was significantly correlated with AF (β = 0.6552, 95% CI [0.0400, 1.2705], *p* < 0.05) DSST (β = 2.2503, 95% CI [0.7231, 3.7775], *p* < 0.01) and composite z-score (β = 0.2926, 95% CI [0.0615, 0.5236], *p* < 0.05), with the opposite trend shown in Model 1. In contrast to the low SUA group, cognitive enhancement was more notable by an increase in SUA (*p* < 0.05). Additionally, in Model 2, the association between SUA and cognitive function was not statistically significant.

**Table 2:** Association between SUA (μmol/L) and cognitive function.

	CERAD			Animal Fluency Test			Digit Symbol Substitution Test			Composite z-score		
	Model 1^1^	Model 2	Model 3	Model 1	Model 2	Model 3	Model 1	Model 2	Model 3	Model 1	Model 2	Model 3
	β (95% CI)	β (95% CI)	β (95% CI)	β (95% CI)	β (95% CI)	β (95% CI)	β (95% CI)	β (95% CI)	β (95% CI)	β (95% CI)	β (95% CI)	β (95% CI)
N	2767	2767	2498	2767	2767	2498	2767	2767	2498	2767	2767	2498
SUA (μmol/L)	−0.0043 (−0.0071, −0.0016) ***	0.0002 (−0.0024, 0.0029)	0.0029 (−0.0002, 0.0059) *	−0.0010 (−0.0034, 0.0015)	0.0001 (−0.0022, 0.0025)	0.0028 (0.0002, 0.0055) **	−0.0160 (−0.0233, −0.0087) ***	−0.0034 (−0.0098, 0.0030)	0.0070 (0.0005, 0.0135) **	−0.0018 (−0.0028, −0.0007) ***	−0.0001 (−0.0011, 0.0008)	0.0014 (0.0004, 0.0023) ***
SUA categories												
Q1(178.4–279.5 μmol/L)	Reference	Reference	Reference	Reference	Reference	Reference	Reference	Reference	Reference	Reference	Reference	Reference
Q2(279.6–333.0 μmol/L)	−0.2486 (−0.9084, 0.4112)	0.1722 (−0.4365, 0.7808)	0.1943 (−0.4522, 0.8408)	0.2121 (−0.3724, 0.7967)	0.2631 (−0.2781, 0.8042)	0.3652 (−0.1902, 0.9206)	−1.4281 (−3.1593, 0.3030)	−0.3363 (−1.8059, 1.1334)	0.2961 (−1.0825, 1.6747)	−0.0817 (−0.3322, 0.1687)	0.0551 (−0.1596, 0.2698)	0.1138 (−0.0948, 0.3223)
Q3(333.1–392.5 μmol/L)	−0.6427 (−1.3142, 0.0288) *	0.1037 (S0.5291, 0.7364)	0.2015 (−0.4789, 0.8819)	0.2253 (−0.3696, 0.8202)	0.2468 (−0.3157, 0.8093)	0.4637 (−0.1208, 1.0481)	−1.4191 (−3.1809, 0.3428)	0.5459 (−0.9819, 2.0737)	1.3182 (−0.1326, 2.7690)	−0.1391 (−0.3939, 0.1158)	0.0927 (−0.1304, 0.3159)	0.1919 (−0.1919, 0.4114) *
Q4(392.6–701.9 μmol/L)	−1.0583 (−1.7305, −0.3861) ***	−0.1951 (−0.8317, 0.4415)	0.2813 (−0.4349, 0.9975)	−0.0182 (−0.6137, 0.5774)	0.0821 (−0.4839, 0.6480)	0.6552 (0.0400, 1.2705) **	−2.3357 (−4.0994, −0.5720) ***	−0.0350 (−1.5720, 1.5021)	2.2503 (0.7231, 3.7775) ***	−0.3005 (−0.5556, −0.0454) **	−0.0170 (−0.2415, 0.2075)	0.2926 (0.0615, 0.5236) **
P trend	0.001	0.525	0.464	0.977	0.804	0.039	0.014	0.765	0.002	0.020	0.966	0.011

1 Model 1: no covariates were adjusted. Model 2: age, gender, and race were adjusted. Model 3: age, gender, race, education, marital status, ratio of family income to poverty, albumin, creatinine, body mass index, diabetes, heart failure, stroke, hypertension, alcohol consumption, and physical activity were adjusted. In the subgroup analysis stratified by age, gender, and race, the model is unadjusted for age, gender, or race, respectively.

* *p* < 0.1;

** *p* < 0.05;

*** *p* < 0.01.

**Figure 2:**
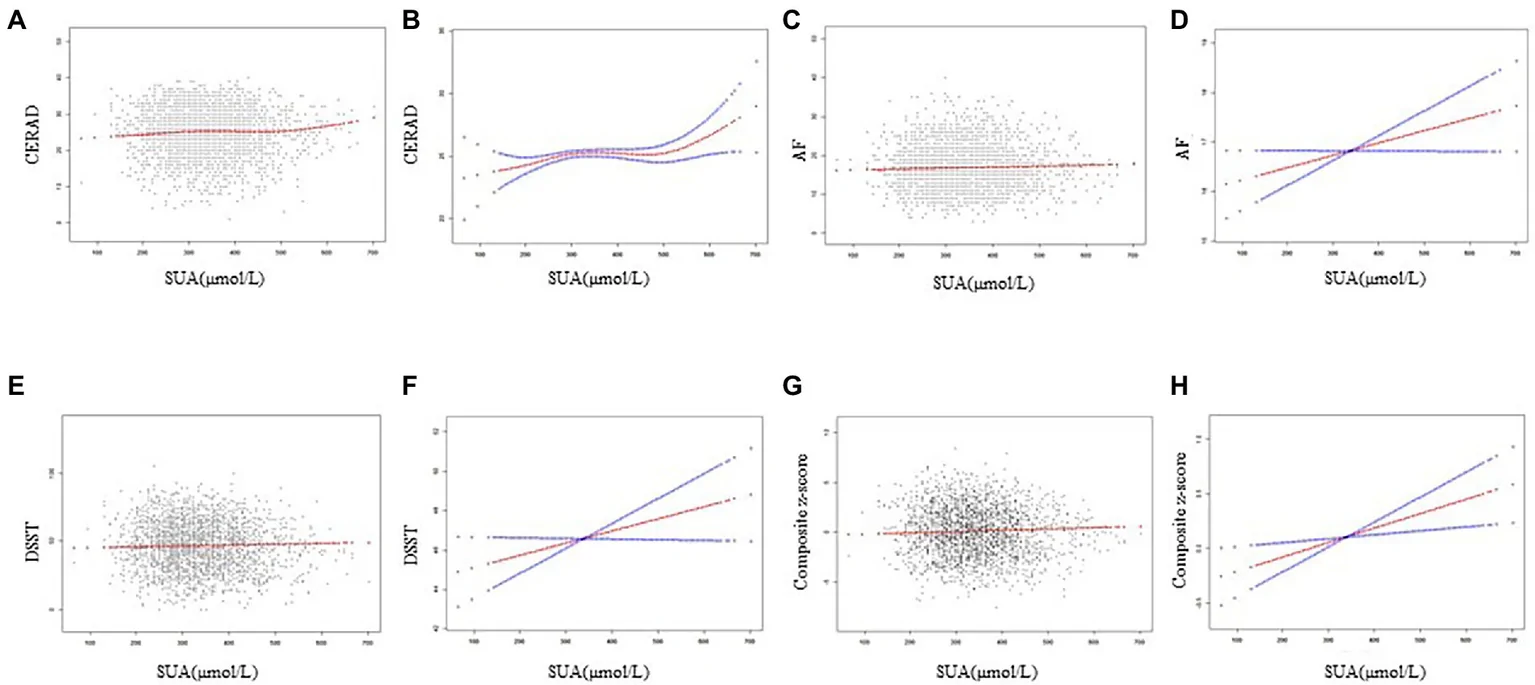
Correlation between SUA and cognitive performance. **(A,C,E,G)** Each black dot represents a sample. **(B,D,F,H)** Solid lines indicate smoothed curve fits between variables. The blue bars indicate the 95% confidence interval of the fit.

Figure 2 depicts the generalized additive models, and smooth curve fitting used to explain the nonlinear association between SUA and cognitive performance. SUA levels and cognitive scores on the AF, DSST, and composite z-score showed positive linear relations. In contrast, a multisegmented linear regression model revealed that for CERAD scores, SUA levels had a curvilinear association with inflection points from 243.9–571.0 μmol/L (Table 3). From 243.9 to 571.0 μmol/L, the trend was flat, while in the remaining range, there was a positive correlation between SUA and CERAD scores. Table 3 demonstrates that there are significant positive connections on either side, while the central flat segment is not statistically significant. The connection between SUA and CERAD is therefore ambiguous and requires further investigation.

**Table 3:** Threshold effect of blood SUA on CERAD score using a multi-piecewise linear regression model.

CERAD Score	Fitting by the standard linear model	Fitting by the two-piecewise linear model	Inflection point	<243.9 μmol/L	243.9–571.0 μmol/L	>571.0 μmol/L	Log likelihood ratio
Adjusted β (95% CI), *p* value	0.003 (−0.000, 0.006), 0.0652		243.9, 571.0	0.015 (−0.001, 0.030), 0.0638	0.000 (−0.003, 0.004), 0.8820	0.081 (0.013, 0.149), 0.0202	<0.001

Age, gender, race, education, marital status, ratio of family income to poverty, albumin, creatinine, body mass index, diabetes, heart failure, stroke, hypertension, alcohol consumption, and physical activity were adjusted.

### Subgroup analyses

To identify potential interaction effects with subgroups, we also assessed the relations between SUA and cognitive function independently by age, gender, and race.

**Figure 3:**
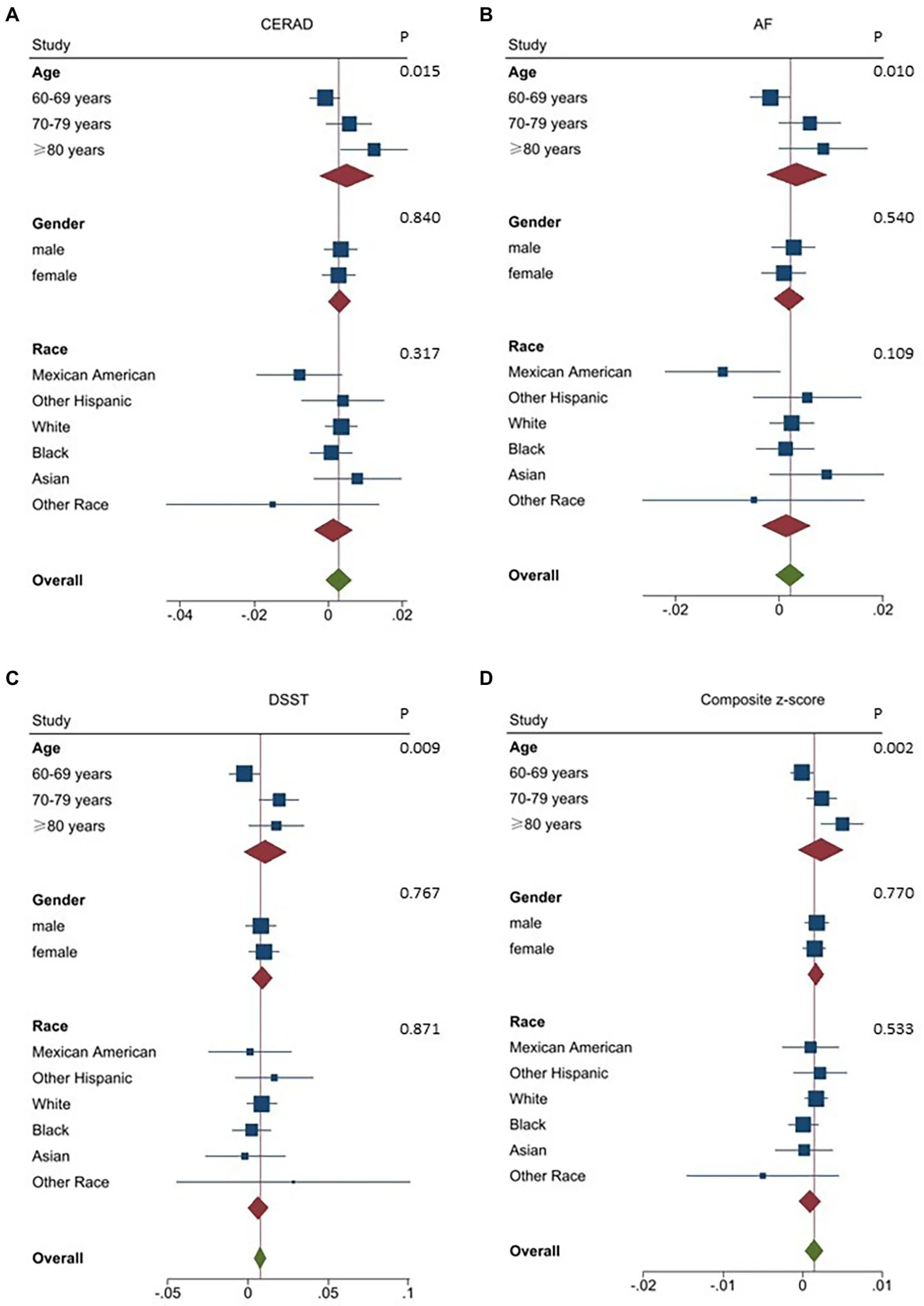
Association between SUA and various domains of cognitive function stratified by demographic characteristics. Age, gender, race, education, marital status, ratio of income to poverty, albumin, creatinine, body mass index, diabetes, heart failure, stroke, hypertension, alcohol consumption, and physical activity were adjusted. In the subgroup analysis stratified by age, gender, and race, the model is not adjusted for age, gender, or race, respectively. **(A)** Association between SUA and CERAD stratified by demographic characteristics.**(B)** Association between SUA and AFstratified by demographic characteristics. **(C)** Association between SUA and DSST stratified by demographic characteristics. **(D)** Association between SUA and Composite z-score stratified by demographic characteristics. CERAD: Consortium to Establish a Registry for Alzheimer’s Disease; AF: Animal fluency test; DSST: Digit symbol substitution test.

With respect to all cognitive test scores (*p* < 0.05; Figure 3), potential interaction effects between SUA and age were found. In participants aged 60–69 years, SUA levels displayed a negative linear relation, with cognitive function declining as SUA levels rose (Model 1: CERAD β = −0.0070, 95% CI [−0.0104, −0.0036], *p* < 0.01; DSST β = −0.0160, 95% CI [−0.0255, −0.0064], *p* < 0.01; composite z-score β = −0.0021, 95% CI [−0.0034, −0.0007], *p* < 0.01; Supplementary Table 1). After controlling for other confounding factors, SUA was found to be positively correlated with both DSST and composite z-score for those aged 70–79 years (DSST β = 0.0194, 95% CI [0.0073, 0.0316], *p* < 0.01; composite z-score β = 0.0024, 95% CI [0.0005, 0.0043], *p* < 0.05; Supplementary Table 1). The same trend was detected for the strong correlation between SUA levels and all cognitive tests among participants aged ≥80 years (CERAD β = 0.0124, 95% CI [0.0035, 0.0214], *p* < 0.01; AF β = 0.0113, 95% CI [0.0055, 0.0170], *p* < 0.01; DSST β = 0.0177, 95% CI [0.0009, 0.0345], *p* < 0.05; composite z-score β = 0.0050, 95% CI [0.0024, 0.0076], *p* < 0.01; Supplementary Table 1).

Sex, race, and SUA levels did not interact on any domain-specific cognitive function or composite z-score in the fully adjusted models (*p* > 0.05; Figures 3A–D).

## Discussion

### Research findings and clinical significance

To analyze the associations between SUA and cognition, we combined NHANES 2011–2012 and 2013–2014 data, which included 2,767 Americans aged ≥60 years. These analyses showed that SUA is significantly correlated with cognitive test scores. For specific dose effects, the results were inconsistent. SUA level was positively correlated with all cognitive test scores, as shown by the smooth curve fitting. The improvement in cognitive function with rising SUA level supports SUA’s preventive role against cognitive decline. Age also had an impact on the association between SUA and cognition. Older adults aged 60–69 exhibited a negative correlation, while those 70 and older showed a positive correlation. SUA was not significantly associated with any cognitive test scores in analyses that were adjusted for gender and race.

In sum, this population’s cognitive and SUA levels were significantly correlated. A rise in SUA level was related to improved cognitive performance across many cognitive areas. High SUA in the memory, language, and digital cognitive domains continued to be associated with increased cognitive function. We further discovered that those over age 70 benefited more from the cognitive protective effects of higher SUA.

Hyperuricemia is indicated by SUA levels >420 μmol/L, which increases the risk of gout attacks. Patients with gout are now typically advised to use long-term SUA-lowering medication to reduce their number of bouts. For most participants with gout, the target SUA value is <360 μmol/L, but for those with a high SUA burden (e.g., tophaceous gout) a SUA value of ≤300 μmol/L may be required (Dalbeth et al., 2021). Our results show that SUA plays a significant role in cognitive impairment. Thus, we suggest improving the objective of SUA management in older patients with gout while still preventing gout attacks. Some anti-inflammatory medications, like the IL-1β inhibitor canakinumab, may avoid gout episodes without changing serum urate concentrations (Dalbeth et al., 2021).

From a dementia prevention perspective, SUA management is unnecessary for healthy individuals. The most up-to-date authoritative literature does not advise using SUA-lowering medications for patients without gout (Dalbeth et al., 2021). Dementia is influenced by various factors, several of which also contribute to preventing cognitive impairment (e.g., improving nutrition, adequate exercise).

### Comparison with previous studies

The overall positive trend between SUA and cognitive performance indicated superior cognitive performance in the older group as SUA levels increased. High SUA levels are advantageous for memory, language, processing speed, attention, and general cognition, consistent with previous research (Lu et al., 2016; Liu et al., 2017; Tana et al., 2018; Boccardi et al., 2021; Zhou et al., 2021; Lee et al., 2021b). The ReGAl 2.0 project (Boccardi et al., 2021), an Italian multicenter clinical trial with 232 participants, showed that SUA is an independent risk factor for dementia, evidenced by a decrease in serum SUA in patients with AD (*p* = 0.001). In addition, a meta-analysis (Zhou et al., 2021) of 53 studies found that participants with dementia had lower SUA levels compared with those without dementia (SMD = −0.32 [−0.64, −0.01] *p* = 0.04).

Some research has suggested that increased SUA could exacerbate cognitive impairment, which is contrary to our findings. Different conclusions were drawn from a study of patients with diabetes. A clinical investigation by Huang et al. (2019) showed a U-shape relation between SUA and the chance of developing MCI in patients with type 2 diabetes; those whose SUA levels were below the cutoff (388.63 μmol/L) had a considerably lower risk of developing MCI with rising SUA levels. These outcomes were reversed for SUA levels above the cutoff. Similarly, a cohort study discovered that high SUA levels may raise the incidence of dementia in older adults, particularly vascular or mixed dementia (Latourte et al., 2018). This discrepancy may be influenced by comorbidities. As a metabolite, SUA also carries a risk of contributing to development of diabetes (Lu et al., 2020), and diabetes increases the risk of dementia (Biessels and Despa, 2018). It has also been demonstrated that hyperuricemia is a risk factor for cerebrovascular (Zhu et al., 2022) and cardiovascular illnesses (Mouradjian et al., 2020). Further, vascular risk factors have been linked to an increased risk of dementia (Gottesman et al., 2017). To protect cognitive function, we should therefore strike a balance between raising uric acid levels within the normal range, while avoiding hyperuricemia from triggering other comorbidities that could eventually lead to dementia.

### Demographic factor effects

Our findings indicate that the connection between SUA and cognition is significantly influenced by age. Individuals aged 70 years and older demonstrated improved cognitive performance when SUA levels increased, after controlling for all variables. A statistically significant association was also discovered among those in the 60–69 year age range, though with a trend in the opposite direction. Other potential demographic factors, including gender and race, had no discernible impact on the relation between SUA and cognition.

Age is one factor that leads to increased SUA (Dehlin et al., 2020; Buzas et al., 2021). Another cross-sectional study revealed that whereas SUA levels in men decline with age, they increase dramatically in women (Sun et al., 2019). However, the ReGAl 2.0 experiment showed no conclusive link between SUA levels and age (Boccardi et al., 2021). Age is also known to increase the risk of dementia and deterioration in cognitive function (Jia et al., 2020). SUA was favorably connected with cognitive performance in the older group herein, suggesting that SUA may ameliorate the cognitive loss brought on by aging. The connection between SUA and age remains unclear, and results among related studies have been inconsistent. Further prospective research on middle-aged and older individuals will be required to evaluate these relations.

### Pathogenic mechanism

SUA, referred to as an essential physiological molecule, has been linked to several illnesses, including gout (Dalbeth et al., 2021), cardiovascular disease (Chang et al., 2018), diabetes (Lu et al., 2020), kidney disease (Hahn et al., 2017), and metabolic syndrome (Yanai et al., 2021). The exact mechanisms by which SUA impacts cognition remain unknown. SUA is a crucial endogenous antioxidant (El Ridi and Tallima, 2017; Yeung et al., 2019) that removes some reactive nitrogen and oxygen compounds, reducing oxidative damage. It has also been shown to minimize oxidative stress (Yeung et al., 2019; Dom Nguez-Zambrano et al., 2020) and protect the nervous system (Ya et al., 2018). SUA also improves dementia by lowering Aβ and tau toxicity. Higher SUA levels significantly reduce the adverse effects of amyloid 1–42 and tau on cognition in women, according to a longitudinal study (Ye et al., 2016). Additionally, hyperuricemia is a well-known indicator of good nutritional status (Dom Nguez-Zambrano et al., 2020); since it is also common for patients with dementia to experience malnutrition (Kimura et al., 2019), SUA may also impact cognitive performance through nutrient metabolism.

Other mechanisms may also help explain why elevated SUA worsens cognitive impairment. Evidence suggests that the neutrophil oxidative burst involves new oxidant urate hydrogen peroxide, which is formed under inflammatory circumstances, changes redox homeostasis, and fosters an oxidative environment (Silva et al., 2018). SUA can be oxidized by peroxidase to produce the potent oxidant urate hydrogen peroxide in the presence of pre-existing neuroinflammation, which further encourages the neuroinflammatory response and oxidative stress, and worsens the pathophysiological alterations of dementia.

### Deviation analysis

Previous studies of the association between SUA and cognition have shown conflicting results. These disputed findings may be explained by sample, study methods, varying cognitive assessment scales, population distributions, and interference by confounding factors. Additionally, many older adults have concurrent medical problems (Silva et al., 2019; Jia et al., 2020; e.g., malnutrition (Kimura et al., 2019), diabetes (Burillo et al., 2021), hypertension [Iadecola and Gottesman, 2019), renal failure (Shi et al., 2018)], all of which have the potential to both alter SUA and impact cognitive function. Furthermore, past studies have revealed a wide range of issues among cognitive rating scales; when cognitive performance is assessed using several cognitive function tests of diverse areas, the association between SUA and cognitive function may also appear erratic.

A meta-analysis (Zhou et al., 2021) to determine whether low SUA is a risk factor for dementia, it was shown to be a potential risk factor for AD and Parkinson’s disease with dementia, but not VAD. Thus, those with different dementia subtypes may also show varying relations between SUA and cognition. Another significant potential source of bias is that the dementia diagnosis itself is prone to error, and frequently constrained by clinician experience; occasionally only a provisional diagnosis of dementia or cognitive decline is made, without a detailed explanation of dementia subtype (Amjad et al., 2018).

### Strengths and limitations

First, inclusion of a large sample of older adults is a major study strength. Second, a comprehensive analysis was made of multiple demographic variables that may impact uric acid and cognitive performance. Third, these analyses offer fresh perspectives on how to manage uric acid levels, and how to prevent and treat clinical cognitive impairment in older adults. We also acknowledge the study’s shortcomings. First, these analyses were based on an American database, and thus may need more relevant studies of older adults in other regions to verify the generalizability of the findings. Second, some confounds could not be completely eliminated; for example, the model did not account for the impacts of diet and medicine. Third, our research was cross-sectional and thus only associations between SUA and cognitive function could be shown. To better understand SUA’s dose-time effects on cognition, prospective studies will be needed. Future studies should also avoid other confounding factors, clarify dementia subtypes, minimize the effects of comorbidities on results, and assess as many cognitive domains as possible. Inclusion of middle-aged participants would help further define the function of age, by observing changes in both SUA and cognitive levels to clarify the timing of changes and demonstrate causal relations.

## Conclusion

Herein, SUA and general cognition were shown to be strongly and positively related. Based on these findings, increasing SUA levels may enhance cognitive function across many cognitive domains. Further, age substantially impacts the association between SUA and cognitive function: elevated SUA has a protective effect on cognition in those over age 70 years. By evaluating the relation between SUA and cognitive performance, our results indicate that high SUA has a cognitive protective effect on older Americans; these cumulative findings offer fresh insights into potential clinical dementia prevention and therapy, and highlight potential SUA management in the study of cognitive impairment. We propose that older adults can appropriately increase uric acid within the normal range to safeguard cognitive function, when used in conjunction with clinical practice. However, hyperuricemia can easily increase the risk of common comorbidities in older adults, including cardiovascular and cerebrovascular illnesses, diabetes, and kidney diseases. It is therefore essential to identify a balancing point to control uric acid in clinical practice, prevent the risk of hyperuricemia, and fully exploit the cognitive protective impacts of uric acid. We also advise against very stringent uric acid control in older patients with gout, as it may be advantageous to maintain their uric acid within the normal range to avoid cognitive damage.

## Data availability statement

The datasets presented in this study can be found in online repositories. The names of the repository/repositories and accession number(s) can be found at: https://wwwn.cdc.gov/nchs/nhanes/continuousnhanes/default.aspx?BeginYear=2011, https://wwwn.cdc.gov/nchs/nhanes/continuousnhanes/default.aspx?BeginYear=2013.

## Author contributions

RG: data collection, analysis, and interpretation. RG, YZ, and ML: study conception and design, preparation of the manuscript, and manuscript review. The manuscript was critically revised by YZ, ML, SD, JD, and HZ for essential intellectual content. QT: accountable for the correctness of data analysis and the integrity of the data. All authors contributed to the article and approved the submitted version.

## Funding

This research was supported by the Guangdong Precision Medicine Foundation (GDPMAA-P-C-2021-009).

## Conflict of interest

The authors declare that the research was conducted in the absence of any commercial or financial relationships that could be construed as a potential conflict of interest.

## Correction note

A correction has been made to this article. Details can be found at: 10.3389/fnagi.2026.1972629.

## Publisher’s note

All claims expressed in this article are solely those of the authors and do not necessarily represent those of their affiliated organizations, or those of the publisher, the editors and the reviewers. Any product that may be evaluated in this article, or claim that may be made by its manufacturer, is not guaranteed or endorsed by the publisher.

## Footnotes

^1^ https://www.r-project.org/

^2^ http://www.empowerstats.net/analysis/
