# Correction: Elevated serum uric acid is associated with cognitive improvement in older American adults: a large, population-based-analysis of the NHANES database

**DOI:** 10.3389/fnagi.2026.1972629

**Published:** 2026-09-21

**Authors:** Rulin Geng, Yu Zhang, Miao Liu, Shengfeng Deng, Jingwen Ding, Hongfei Zhong, Qiuyun Tu

**Affiliations:** Department of Geriatrics, The Fifth Affiliated Hospital of Sun Yat-sen University, Zhuhai, China

**Keywords:** cognitive function, dementia, Alzheimer's disease, serum uric acid, NHANES

There was a mistake in Table 3 as published. In the published version, several numerical values from an earlier analytical version were inadvertently concatenated with the final revised values in Table 3. This concatenation was not present in the author-approved proof, which contained only the final revised numerical results. In addition, the upper-threshold column heading was incorrectly presented as “SUA >505.2 μmol/L”; the correct heading is “SUA >571.0 μmol/L”, consistent with the final inflection points of 243.9 and 571.0 μmol/L and the Results section. The corrected Table 3 appears below. These corrections do not involve reanalysis of the underlying data and do not affect the results, statistical interpretation, or conclusions of the article.

**Table 3:** Threshold effect of blood SUA on CERAD score using a multi-piecewise linear regression model.

CERAD score	Fitting by the standard linear model	Fitting by the two-piecewise linear model	Inflection point	SUA			Log likelihood ratio
				<**243.9** μ**mol/L**	**243.9–571.0** μ**mol/L**	>**571.0** μ**mol/L**	
Adjusted β (95% CI), *P*-value	0.003 (−0.000, 0.006), 0.0652		243.9, 571.0	0.015 (−0.001, 0.030), 0.0638	0.000 (−0.003, 0.004), 0.8820	0.081 (0.013, 0.149), 0.0202	< 0.001

Age, gender, race, education, marital status, ratio of family income to poverty, albumin, creatinine, body mass index, diabetes, heart failure, stroke, hypertension, alcohol consumption, and physical activity were adjusted.

The original version of this article has been updated.

