# Supplementary material for: Elevated serum uric acid is associated with cognitive improvement in older American adults: A large, population-based-analysis of the NHANES database
Source: Front Aging Neurosci. 2022 Dec 8;14:1024415. doi: 10.3389/fnagi.2022.1024415 (PMC9772611; doi:10.3389/fnagi.2022.1024415)
Supplement: Supplementary file 1 [file Table_1.docx]

Supplement Table 1. The association between uric acid and cognitive function, stratified by age, gender and race

|  | CERAD | | | AF | | | | DSST | | | | Composite z-score | | | |  |
| --- | --- | --- | --- | --- | --- | --- | --- | --- | --- | --- | --- | --- | --- | --- | --- | --- |
|  | Model 1^1^  β (95% CI) | Model 2  β (95% CI) | Model 3  β (95% CI) | | Model 1  β (95% CI) | Model 2  β (95% CI) | Model 3  β (95% CI) | | Model 1  β (95% CI) | Model 2  β (95% CI) | Model 3  β (95% CI) | | Model 1  β (95% CI) | Model 2  β (95% CI) | Model 3  β (95% CI) | |
| Age |  |  |  | |  |  |  | |  |  |  | |  |  |  | |
| 60-69 years | -0.0070 (-0.0104, -0.0036) *** | -0.0027 (-0.0062, 0.0008) | -0.0008 (-0.0048, 0.0032) | | -0.0004 (-0.0038, 0.0029) | -0.0002 (-0.0036, 0.0032) | 0.0009 (-0.0029, 0.0046) | | -0.0160 (-0.0255, -0.0064) *** | -0.0012 (-0.0104, 0.0080) | -0.0023 (-0.0115, 0.0069) | | -0.0021 (-0.0034, -0.0007) *** | -0.0005 (-0.0018, 0.0008) | -0.0001 (-0.0015, 0.0013) | |
| 70-79 years | -0.0003 (-0.0054, 0.0048) | 0.0026 (-0.0025, 0.0076) | 0.0057 (-0.0004, 0.0118) * | | -0.0008 (-0.0053, 0.0036) | -0.0004 (-0.0048, 0.0040) | 0.0023 (-0.0029, 0.0075) | | -0.0118 (-0.0241, 0.0005) * | -0.0045 (-0.0159, 0.0069) | 0.0194 (0.0073, 0.0316) *** | | -0.0009 (-0.0027, 0.0009) | 0.0001 (-0.0017, 0.0018) | 0.0024 (0.0005, 0.0043) ** | |
| ≥80 years | 0.0040 (-0.0031, 0.0111) | 0.0052 (-0.0018, 0.0123) | 0.0124 (0.0035, 0.0214) *** | | 0.0017 (-0.0030, 0.0064) | 0.0023 (-0.0024, 0.0070) | 0.0113 (0.0055, 0.0170) *** | | -0.0063 (-0.0214, 0.0088) | -0.0028 (-0.0173, 0.0116) | 0.0177 (0.0009, 0.0345) ** | | 0.0006 (-0.0017, 0.0028) | 0.0011 (-0.0011, 0.0033) | 0.0050 (0.0024, 0.0076) *** | |
| Gender |  |  |  | |  |  |  | |  |  |  | |  |  |  | |
| male | 0.0024 (-0.0016, 0.0065) | 0.0009 (-0.0029, 0.0047) | 0.0034 (-0.0010, 0.0078) | | 0.0028 (-0.0012, 0.0067) | 0.0019 (-0.0018, 0.0056) | 0.0043 (0.0001, 0.0085) ** | | 0.0036 (-0.0068, 0.0141) | 0.0006 (-0.0084, 0.0096) | 0.0079 (-0.0014, 0.0172) * | | 0.0011 (-0.0005, 0.0026) | 0.0005 (-0.0008, 0.0019) | 0.0018 (0.0003, 0.0032) ** | |
| female | -0.0045 (-0.0084, -0.0005) ** | -0.0005 (-0.0043, 0.0033) | 0.0028 (-0.0016, 0.0071) | | -0.0054 (-0.0087, -0.0021) *** | -0.0013 (-0.0044, 0.0018) | 0.0026 (-0.0009, 0.0060) | | -0.0217 (-0.0324, -0.0110) *** | -0.0050 (-0.0142, 0.0041) | 0.0099 (0.0004, 0.0193) ** | | -0.0029 (-0.0044, -0.0014) *** | -0.0006 (-0.0019, 0.0007) | 0.0015 (0.0001, 0.0029) ** | |
| Race |  |  |  | |  |  |  | |  |  |  | |  |  |  | |
| Mexican American | -0.0094 (-0.0193, 0.0005) * | -0.0051 (-0.0148, 0.0047) | -0.0078 (-0.0192, 0.0037) | | 0.0072 (-0.0011, 0.0155) * | 0.0082 (0.0000, 0.0165) * | 0.0112 (0.0012, 0.0213) ** | | -0.0149 (-0.0415, 0.0117) | -0.0080 (-0.0343, 0.0184) | 0.0012 (-0.0244, 0.0268) | | -0.0010 (-0.0046, 0.0027) | 0.0003 (-0.0032, 0.0038) | 0.0010 (-0.0025, 0.0045) | |
|  | | | | | | | | | | | | | | | | |
| Supplement Table 1. Cont | | | | | | | | | | | | | | | | |
|  | CERAD | | | AF | | | | DSST | | | | Composite z-score | | | |  |
|  | Model 1^1^  β (95% CI) | Model 2  β (95% CI) | Model 3  β (95% CI) | | Model 1  β (95% CI) | Model 2  β (95% CI) | Model 3  β (95% CI) | | Model 1  β (95% CI) | Model 2  β (95% CI) | Model 3  β (95% CI) | | Model 1  β (95% CI) | Model 2  β (95% CI) | Model 3  β (95% CI) | |
| Other Hispanic | -0.0062 (-0.0151, 0.0026) | -0.0003 (-0.0091, 0.0086) | 0.0040 (-0.0070, 0.0151) | | 0.0006 (-0.0063, 0.0074) | -0.0003 (-0.0074, 0.0069) | 0.0037 (-0.0052, 0.0125) | | -0.0112 (-0.0361, 0.0136) | 0.0065 (-0.0183, 0.0313) | 0.0164 (-0.0080, 0.0408) | | -0.0015 (-0.0047, 0.0016) | 0.0003 (-0.0029, 0.0034) | 0.0022 (-0.0012, 0.0055) | |
| White | -0.0044 (-0.0084, -0.0004) ** | 0.0001 (-0.0037, 0.0039) | 0.0035 (-0.0009, 0.0079) | | -0.0002 (-0.0037, 0.0034) | 0.0004 (-0.0031, 0.0038) | 0.0037 (-0.0001, 0.0075) * | | -0.0150 (-0.0249, -0.0051) *** | -0.0040 (-0.0131, 0.0051) | 0.0085 (-0.0008, 0.0178) * | | -0.0016 (-0.0031, -0.0001) ** | -0.0002 (-0.0015, 0.0012) | 0.0017 (0.0003, 0.0031) ** | |
| Black | -0.0018 (-0.0072, 0.0036) | 0.0009 (-0.0042, 0.0061) | 0.0008 (-0.0049, 0.0065) | | -0.0021 (-0.0065, 0.0022) | -0.0025 (-0.0067, 0.0018) | -0.0009 (-0.0056, 0.0038) | | -0.0057 (-0.0196, 0.0081) | 0.0005 (-0.0119, 0.0130) | 0.0023 (-0.0097, 0.0144) | | -0.0010 (-0.0029, 0.0009) | -0.0003 (-0.0021, 0.0015) | 0.0001 (-0.0017, 0.0019) | |
| Asian | -0.0063 (-0.0172, 0.0046) | -0.0004 (-0.0106, 0.0099) | 0.0079 (-0.0038, 0.0196) | | -0.0081 (-0.0154, -0.0009) ** | -0.0079 (-0.0153, -0.0004) ** | -0.0045 (-0.0136, 0.0046) | | -0.0291 (-0.0561, -0.0020) ** | -0.0281 (-0.0545, -0.0017) ** | -0.0019 (-0.0267, 0.0229) | | -0.0041 (-0.0077, -0.0006) ** | -0.0031 (-0.0065, 0.0003) * | 0.0002 (-0.0033, 0.0038) | |
| Other Race | 0.0055 (-0.0103, 0.0213) | 0.0049 (-0.0129, 0.0227) | -0.0150 (-0.0402, 0.0102) | | 0.0041 (-0.0140, 0.0222) | 0.0006 (-0.0201, 0.0212) | -0.0209 (-0.0588, 0.0170) | | 0.0209 (-0.0139, 0.0557) | 0.0103 (-0.0279, 0.0484) | 0.0284 (-0.0442, 0.1009) | | 0.0028 (-0.0029, 0.0085) | 0.0014 (-0.0049, 0.0078) | -0.0050 (-0.0148, 0.0048) | |

^1^Model 1: no covariates were adjusted. Model 2: age, gender, and race were adjusted. Model 3: Age, gender, race, education, marital status, ratio of income to poverty, albumin, creatinine, body mass index, diabetes, heart failure, stroke, hypertension, alcohol drinking, and physical activity were adjusted. In the subgroup analysis stratified by age, gender, and race, the model is not adjusted for age, gender, and race, respectively. CERAD: Consortium to Establish a Registry for Alzheimer’s Disease; AF: Animal Fluency test; DSST: Digit Symbol Substitution Test* p < 0.1; ** p < 0.05; ***p < 0.01
